# Geometrical structure data of nanoporous carbon systems obtained from computer simulated pyrolysis

**DOI:** 10.1016/j.dib.2019.103874

**Published:** 2019-03-28

**Authors:** Jesús Muñiz, Néstor David Espinosa-Torres, Alfredo Guillén-López, Adriana Longoria, Ana Karina Cuentas-Gallegos, Miguel Robles

**Affiliations:** a Instituto de Energías Renovables, Universidad Nacional Autónoma de México, Priv. Xochicalco s/n, Col. Centro, Temixco, Morelos. CP 62580, Mexico; b CONACYT- Universidad Nacional Autónoma de México, Priv. Xochicalco S/n, Col. Centro, Temixco, Morelos. CP 62580, Mexico

**Keywords:** ReaxFF simulations, Energy storage, Pyrolysis, Nanoporous carbon

## Abstract

This article contains data on nanoporous carbon materials coming from lignocellulosic components. Such data is directly related to the research paper “Insights into the design of carbon electrodes coming from lignocellulosic components pyrolysis with potential application in energy storage devices: A combined *in silico* and experimental study” [1]. In this work, the geometrical parameters of nanoporous carbon systems were found with Molecular Dynamics (MD) simulations at the ReaxFF level. The tridimensional structures of such carbon systems are given in Cartesian coordinates. They were computed at different heating rates, simulating the conditions observed in pyrolysis processes of *Agave angustifolia* leaves, which were carried out in a solar furnace. Nanoporous carbon systems are characterized with radial distribution functions (RDF) and ring distribution profiles.

**Table:** Specifications table

Subject area	*Computational chemistry*
More specific subject area	*Molecular dynamics simulation of pyrolysis of* lignocellulosic materials
Type of data	*Table, graph, figure*
How data was acquired	NVT Molecular Dynamics simulations with the Berendsen thermostat at the ReaxFF level, using LAMMPS simulation code. Data processing using gnuplot and ISAACS software and the molecular viewer VMD and VESTA
Data format	*Analyzed*
Experimental factors	*The heating ramps performed in a solar furnace for biomass pyrolysis, were considered in the acquisition of these data. The data obtained with our computer simulations may directly be compared to experimental data.*
Experimental features	
Data source location	*Instituto de Energías Renovables, Universidad Nacional Autónoma de México, Priv. Xochicalco s/n, Col. Centro, Temixco, Morelos. CP 62580, Mexico.*
Data accessibility	Dataset published on Mendeley Data: (https://doi.org/10.17632/s6vs45j7st.1)
Related research article	J. Muñiz, N.D. Espinosa-Torres, A. Guillén-López, A. Longoria, A.K. Cuentas-Gallegos, M. Robles, Insights into the design of carbon electrodes for energy storage devices coming from lignocellulosic components pyrolysis: A combined *in silico* and experimental study, J. Anal. App. Pyr. (2018) in press [1].

**Table:** 

**Value of the Data** • The nanoporous carbon Cartesian coordinates data may be used as input geometries in the theoretical multiscale modeling of carbon electrodes in energy storage devices. • The data related to radial distribution functions may provide insights into the molecular morphology of lignocellulosic components obtained after a biomass pyrolysis process. • The structural data may be implemented as substrates to identify ion diffusion pathways that are relevant in the understanding of electrochemical energy storage in carbon electrodes coming from pyrolyzed biomass. • The nanoporous carbon tridimensional data may be used as support models to simulate the interactions in heterogeneous catalysis.

## 1 Data

**Fig. 1:**
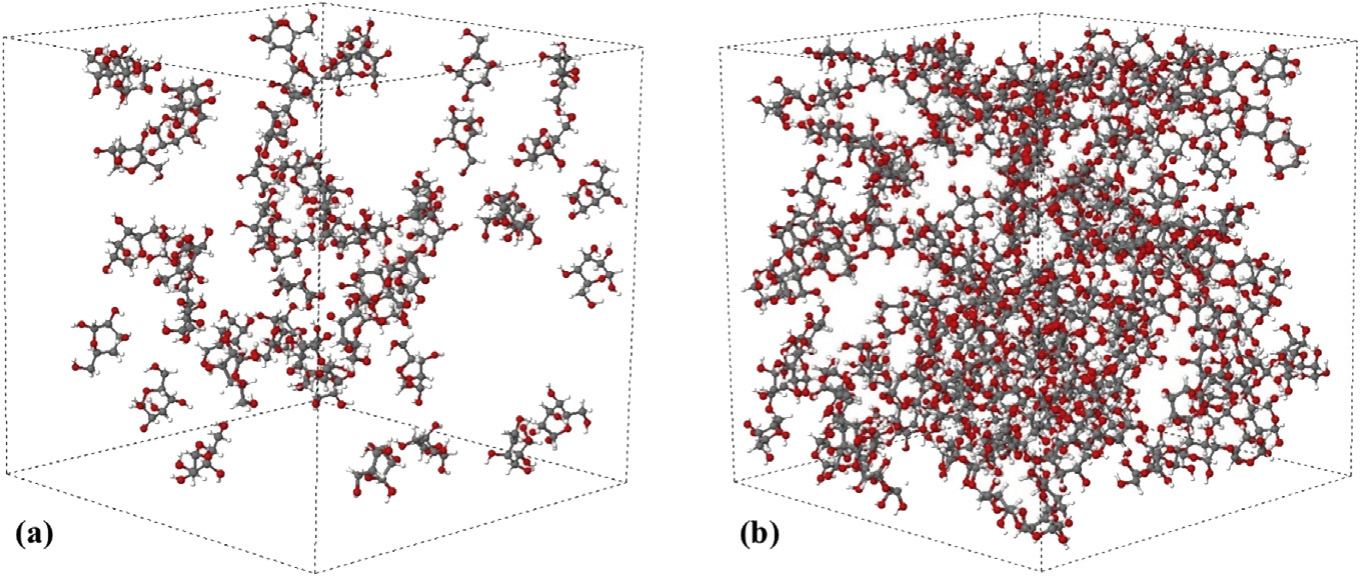
Massive (a) cellulose and (b) hemicellulose model units located at aleatory positions inside a unit cell of 45 Å.

**Fig. 2:**
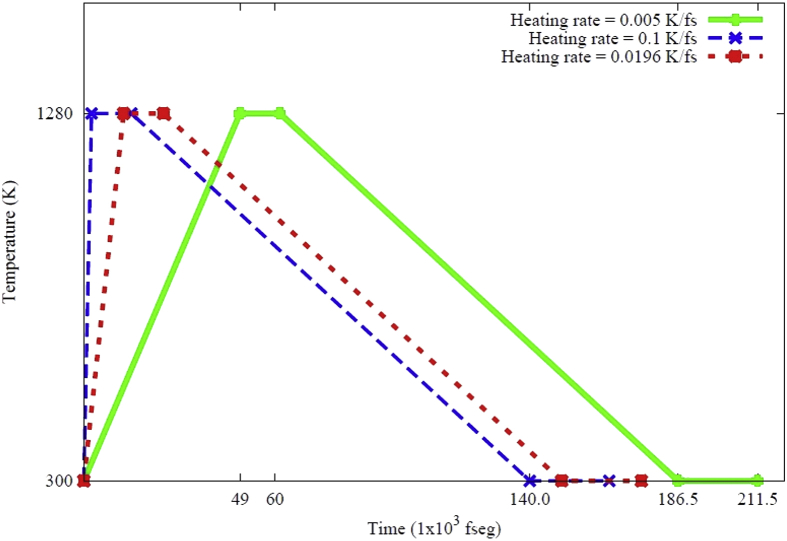
Heating rates used during the simulated pyrolysis processes of lignin, cellulose and hemicellulose. Note that the same rates were implemented for Approaches **1** and **2**.

**Fig. 3:**
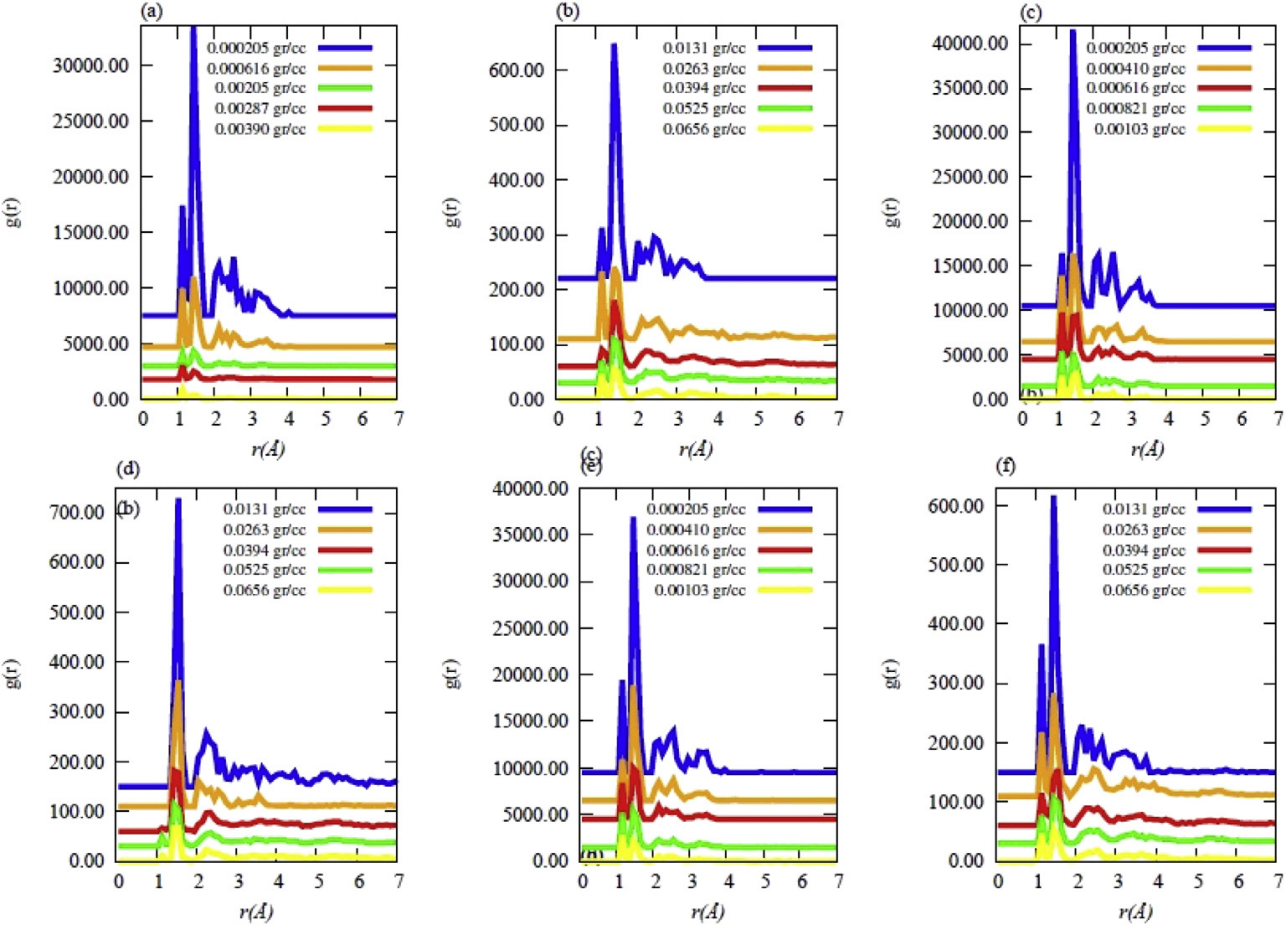
Radial distribution functions (RDF) for the cellulose model at different final densities obtained after simulated pyrolysis for heating rates (HR) of (a),(b) HR = 0.1 K/fs; (c),(d) HR = 0.0196 K/fs; (e),(f) HR = 0.005 K/fs.

**Fig. 4:**
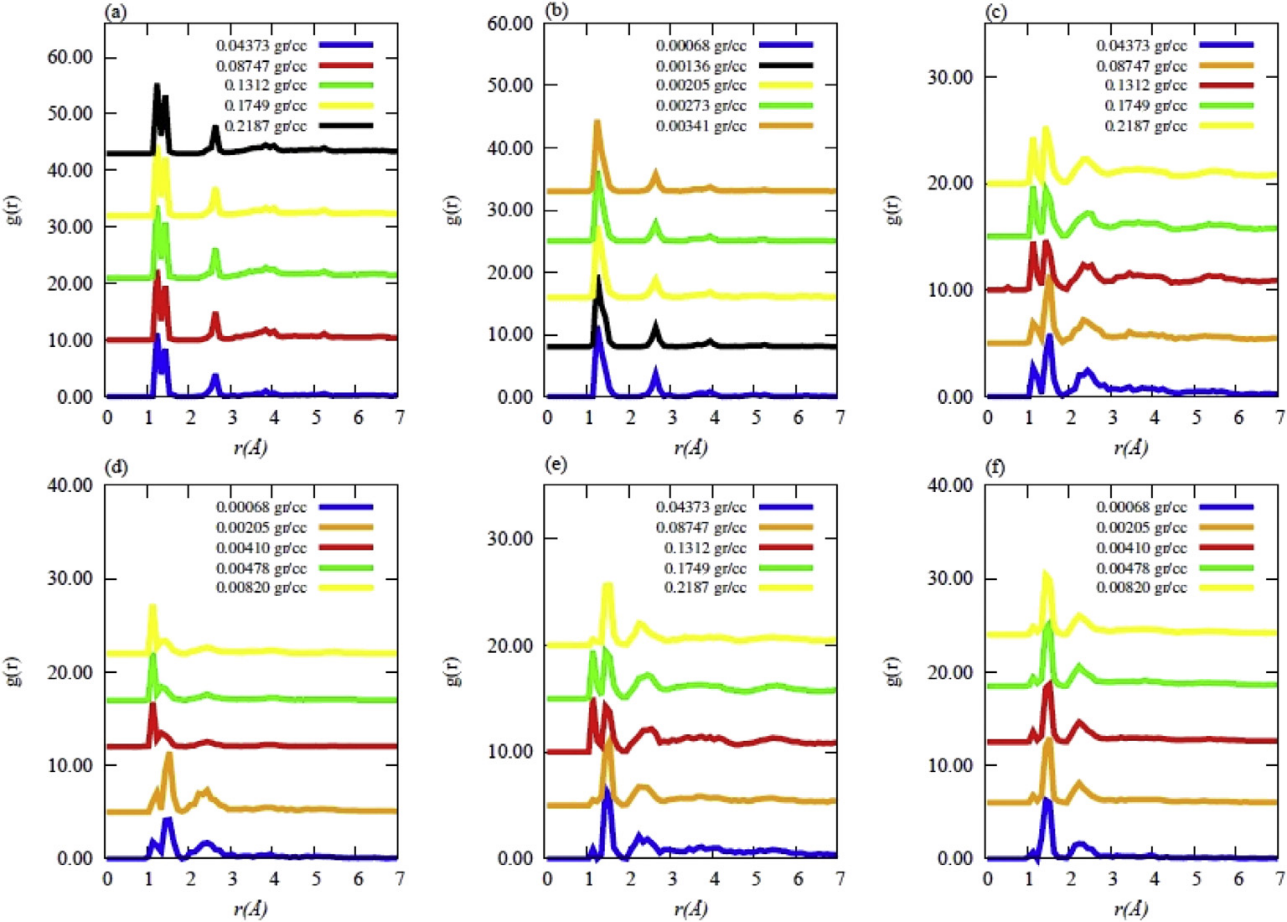
Radial distribution functions (RDF) for the hemicellulose model at different final densities obtained after simulated pyrolysis for heating rates (HR) of (a),(b) HR = 0.1 K/fs; (c),(d) HR = 0.0196 K/fs; (e),(f) HR = 0.005 K/fs.

**Fig. 5:**
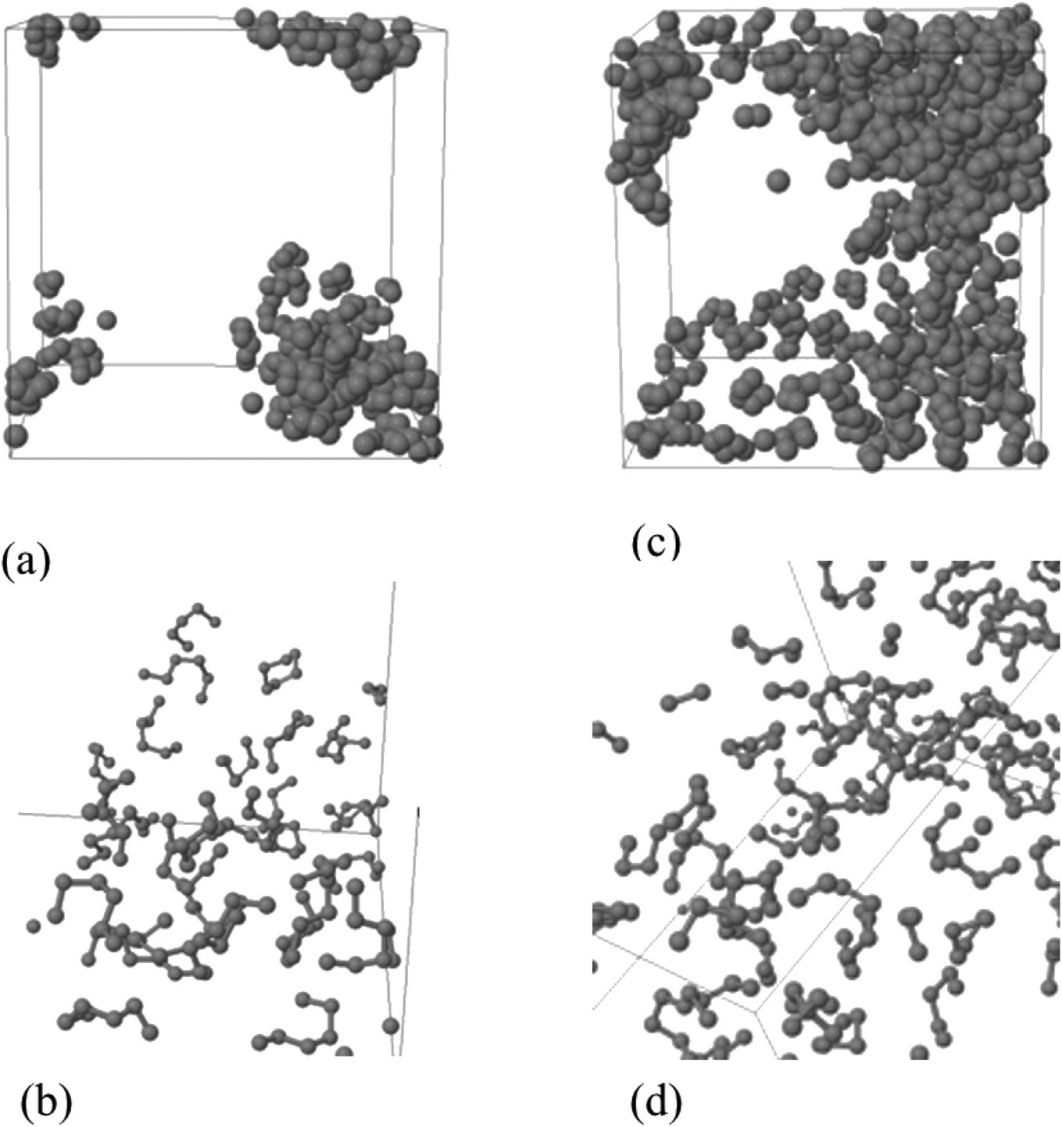
(a) Amorphous carbon structure after cellulose pyrolysis, as obtained at a heating rate of 0.0196 K/fs; (b) Close-up of the carbon structure shown on (a); (c) Amorphous carbon structure after hemicellulose pyrolysis, as obtained at a heating rate of 0.0196 K/fs; (d) Close-up of the carbon structure shown on (c).

**Fig. 6:**
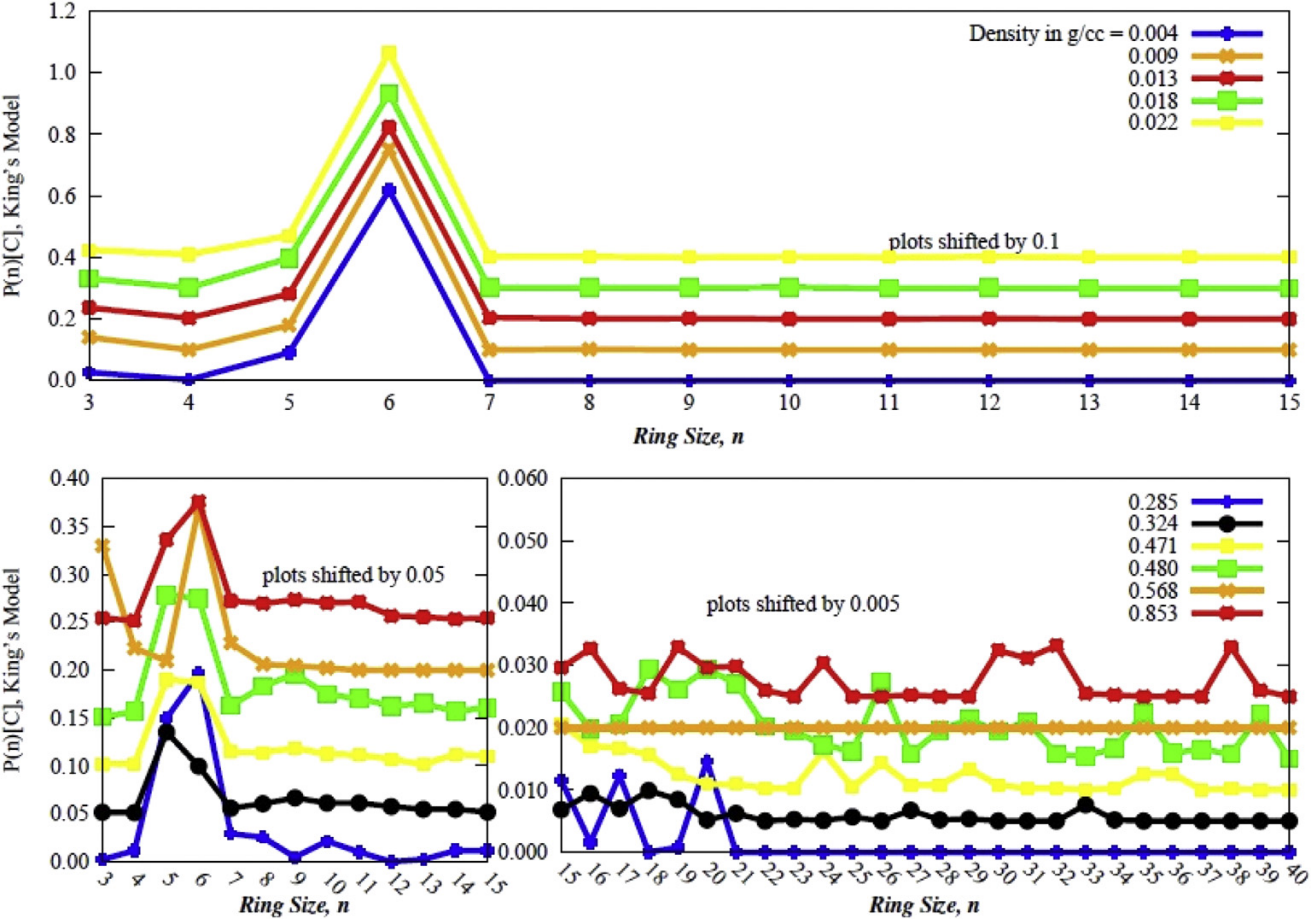
Ring counting on the final pyrolyzed Adler's softwood lignin model for all densities of the data. (a) Ring counting on lower densities; (b) Ring counting on higher densities. Note that the bottom right scale has been broken, since the formation of exotic rings larger than 15-members is less likely to be expected at such densities.

**Fig. 7:**
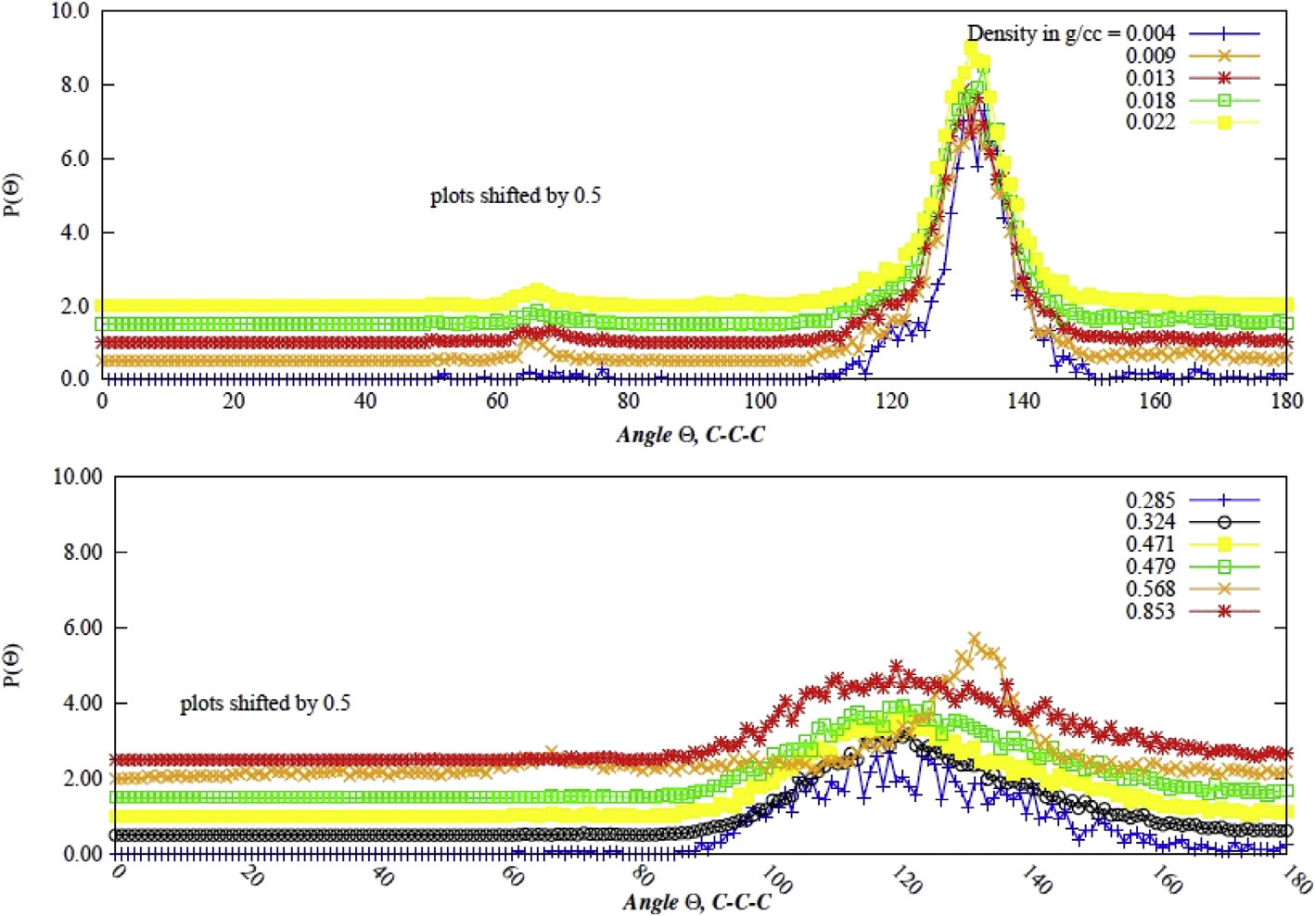
Angle distribution function (P(θ)) for all rings on the final pyrolyzed Adler's softwood lignin model for all densities of the data. (a) P(θ) for lower densities; (b) P(θ) for higher densities.

**Fig. 8:**
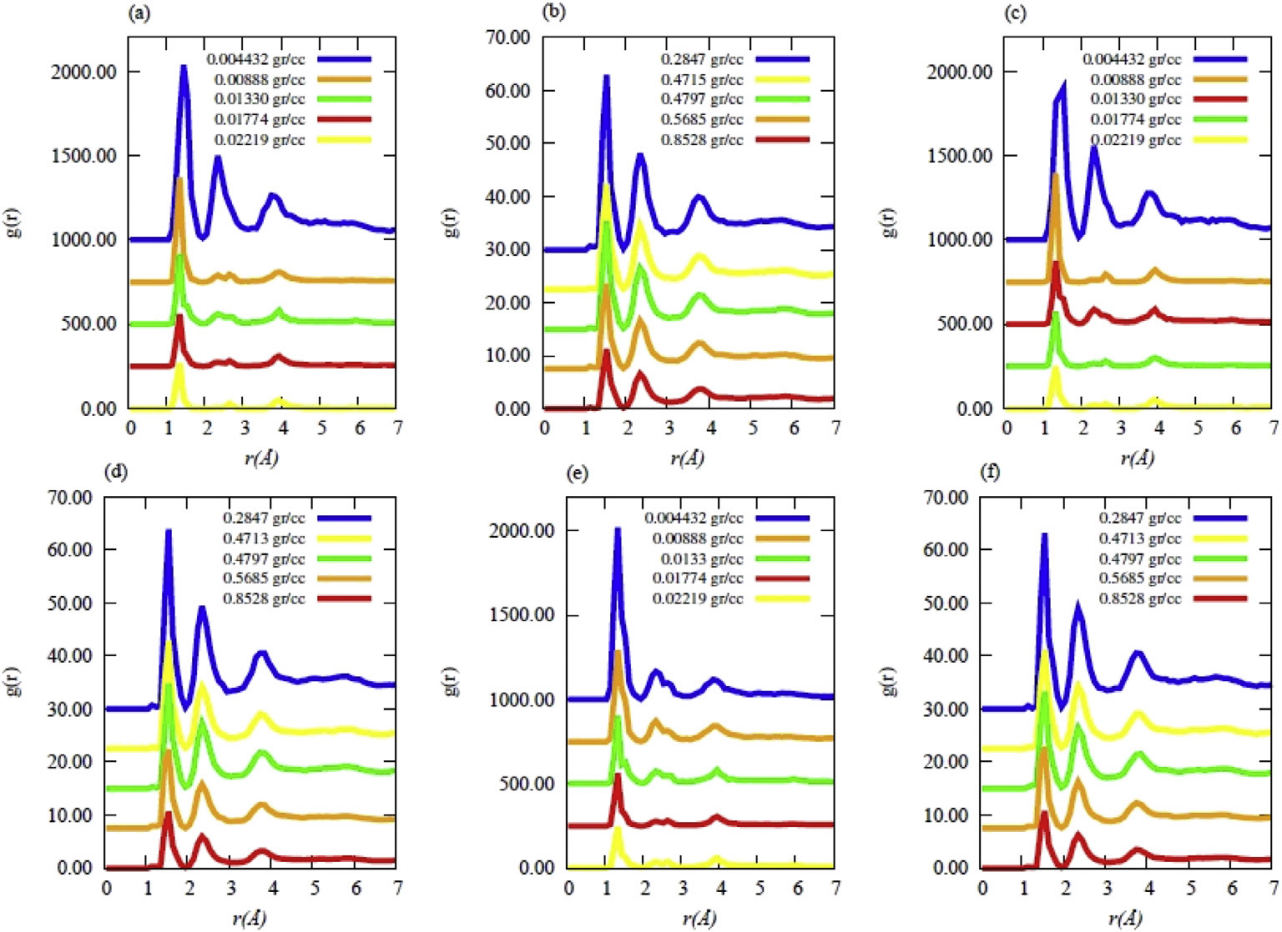
Radial distribution functions (RDF) for the Adler's softwood lignin model at different final densities obtained after simulated pyrolysis for heating rates (HR) of (a),(b) HR = 0.1 K/fs; (c),(d) HR = 0.0196 K/fs; (e),(f) HR = 0.005 K/fs. Note that all simulations were performed using Approach **2**; i.e., oxygen and hydrogen atoms were detached after heating.

**Fig. 9:**
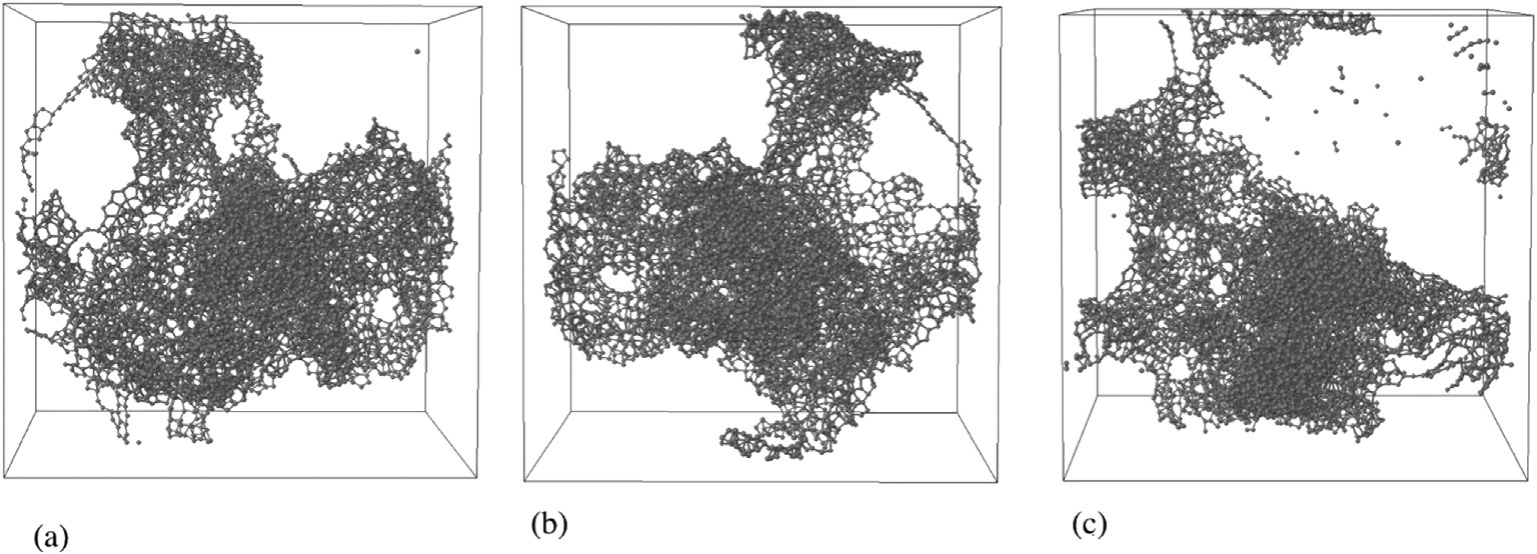
Molecular representations of the simulated char with the final pyrolyzed Adler's softwood lignin model (Approach **2**). The simulated pyrolysis was performed at a density of 0.472 gr/cc with the different heating rates: (a) 0.1 K/fs; (b) 0.0196 K/fs; (c) 0.005 K/fs.

**Fig. 10:**
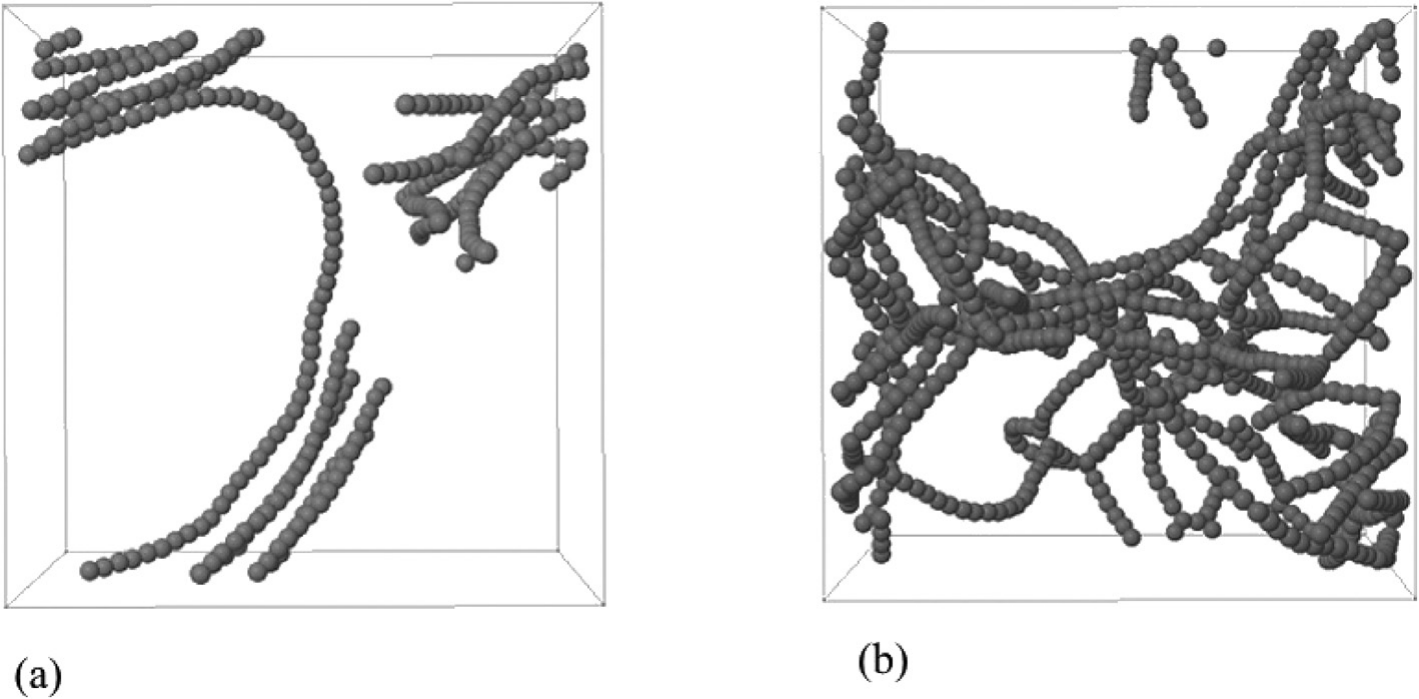
Amorphous carbon structures after pyrolysis as obtained at a heating rate of 0.001 K/fs, (a) cellulose with a density of 0.0657 gr/cc and (b) hemicellulose with a density of 0.219 gr/cc.

Data contains the structural description and at the atomistic level of nanoporous carbon models obtained from simulated pyrolysis processes. Electronic structure properties may be found in the Supplementary Data of the related research article [1]. Calculations were performed at the ReaxFF level with the heating rates of 0.005, 0.0196 and 0.1 K/fs with simulation timing of 211.5, 175.9 and 165.0 × 10^3^ fs, respectively. Pyrolyzed lignin simulations are closely related to experimental data and their Cartesian coordinates are listed as Supplementary Data in the related research article [1] for a total of 62 systems. Fig. 1 shows the massive molecular models of lignocellulosic components and Fig. 2 depicts the specifications of the corresponding heating rates, by showing the evolution of the simulated pyrolysis processes. Data related to the RDF profiles of pyrolyzed cellulosic and hemicellulosic components are presented in Fig. 3, Fig. 4, Fig. 8. Fig. 5, Fig. 10 depict the morphology of cellulose and hemicellulose after the pyrolysis process, while Fig. 9 presents the pyrolyzed morphology of lignin. Fig. 6, Fig. 7 show the ring and angle distribution functions of the nanoporous carbon structures coming from lignin. Finally, Table 1, Table 2 show the parameters used in the Molecular Dynamics simulations at the ReaxFF level. Additionally, the reactive force field used in our Molecular dynamics simulations was included in the Supplementary data of the related research article [1].

**Table 1:** Number of atoms considered in the pyrolysis simulation of lignocellulosic precursors.

Precursors	No. of atoms used in the simulations
Lignin	2700-21600
Cellulose	720-4560
Hemicellulose	750-9000

**Table 2:** Simulation regimes for all lignocellulosic precursors of the data.

Temperature regime (K)	Heating rate (K/fs)	Simulation time (fs)
300-1200-300	0.005	211.5 × 10^3^
300-1200-300	0.0196	175.0 × 10^3^
300-1200-300	0.1	165.0 × 10^3^

## 2 Experimental design, materials, and methods

### 2.1 Data from Density Functional Theory (DFT) calculations

It is important to denote that Density Functional Theory (DFT) data on the magnitude of the atomic charges was calculated for the molecular models of lignin, cellulose and hemicellulose. Such data were presented in Tables S4-S6 of Supplementary Information in the related research article [1]. Such data were obtained using DFT at the PBE/6-31G++ [2] level. Additionally, Natural Bond Order (NBO) analysis [3] was performed to localize the electronic charge at each of the atoms in the lignocellulosic components. Such data are relevant to explore possible reactivity in the lignocellulosic molecules. DFT calculations were performed using Gaussian 09 computational code [4]. The data was obtained from fully relaxed structures, which were performed at the same level of theory given above. DFT data were found in vacuum to simulate the conditions of pyrolysis in a heating pot at a solar furnace [1].

### 2.2 Data from Molecular Dynamics (MD) simulations

The simulated pyrolysis of lignocellulosic components was carried out using MD calculations at the ReaxFF level [5] on a massive model of lignin with the Adler's approach [6]. MD calculations were performed using LAMMPS [7] computational code. Moreover, the cellulose and hemicellulose models were implemented in accordance to Jablan et al. [8], Adel et al. [9] and Zhang et al. [10]. The pyrolyzed systems were nanoporous carbon materials, whose Cartesian coordinates are summarized as Supplementary data in the related research article [1]. Such data were acquired by considering that all components of Adler's lignin model were present during the pyrolysis (Approach 1). Furthermore, Approach 1 was also implemented for the massive model of cellulose and hemicellulose. The initial parameters considered in the computational simulations are all presented in Table 1, Table 2 Data obtained from this pyrolysis are summarized in the RDF profiles of Fig. 3, Fig. 4, respectively.

All MD simulations were performed with the NVT ensemble, and keeping fixed the number N of atoms in a simulation box. In the computations, the equation of motion for all atoms was obtained with a 0.25 × 10^−15^ sec time step. The chemical reactivity through the simulated pyrolysis was modeled with the reactive force field (FF) potential of Kim et al. [11], which is implemented in the ReaxFF [5] module of LAMMPS code [7]. ReaxFF potentials are capable to describe the rising and weakening of chemical bonding with the aid of localized atomic charges. Additionally, ReaxFF introduces the van der Waals (vdW)-type interactions playing a role in the C—C bonding through the simulated pyrolysis. Such attraction is modeled with a distance-corrected Morse potential. In our computations, the vdW interactions were included up to 10 Å. The ReaxFF scheme is also based upon the bond orders (BO) with different atoms to simulate the chemical reactions involved in the pyrolysis processes. Consequently, the total energy of a system modeled with the ReaxFF methodology is described in accordance to:

(1) $$E_{\text{system}}=E_{\text{bond}}+E_{\text{over}}+E_{\text{under}}+E_{\text{lp}}+E_{\text{val}}+E_{\text{tor}}+E_{\text{vdW}}+E_{\text{Coulomb}}$$

in which E_bond_, E_over_, E_under_, E_lp_, E_val_, E_tor_, E_vdW_, E_Coulomb_, correspond to bond energy, overcoordination stability, undercoordination energy, lone pair, valence, torsion, vdW interactions and Coulomb energies, respectively. Additionally, the non-bonding interactions are also contained in the ReaxFF framework, with the introduction of a shielding contribution in the Coulomb and vdW energy terms. Possible discontinuities in the non-bonding energies are avoided with the van Duin's seventh-order taper function [12]. All NVT-MD computations were carried out with a time-step of 0.25 fs by controlling the temperature with the Berendsen [13] thermostat, using a temperature damping of 0.1 ps.

Finally, it was also considered that oxygen and hydrogen were released at a limit temperature of 1280K during the simulation (Approach 2). The RDF profiles for those structural data are summarized in Fig. 8, for the Adler's lignin model. The heating rates in all simulations were performed at 0.005, 0.0196 and 0.1 K/fs, starting from 300K up to 1280K during 196000, 50000 and 9800 steps. A second stage in the simulation was performed with constant temperature of 1280K during 50000 simulation steps. Consequently, a quenching rate was performed by 500000 steps until reaching room temperature. The process was followed up by a stabilization stage at room temperature for 100000 simulation steps. The final densities of the nanoporous structure data range from 0.00443 to 0.853 gr/cc.

The estimation of pressure has previously been performed in accordance to previous methodology [14], in which the pressure was evaluated from the van der Waals equation of state for a pure n-dodecane model system. In the case of lignin components, no van der Waals constants values (*a* and *b*) are available in open literature. The determination of such parameters is out of the scope of the present work and represents a subject of research for future work.
