# Supplementary material for: Geometrical structure data of nanoporous carbon systems obtained from computer simulated pyrolysis
Source: Data Brief. 2019 Mar 28;24:103874. doi: 10.1016/j.dib.2019.103874 (PMC6454095; doi:10.1016/j.dib.2019.103874)
Supplement: Supplementary file 1 — Multimedia component 1 [file mmc1.docx]

**Conflict of interest form**

The authors declare no conflict of interest

The authors

Jesús Muñiz, Néstor David Espinosa-Torres, Alfredo Guillén-López, Adriana Longoria, Ana Karina Cuentas-Gallegos, Miguel Robles

Instituto de Energías Renovables

Universidad Nacional Autónoma de México
